# EnsInfer: a simple ensemble approach to network inference outperforms any single method

**DOI:** 10.1186/s12859-023-05231-1

**Published:** 2023-03-24

**Authors:** Bingran Shen, Gloria Coruzzi, Dennis Shasha

**Affiliations:** 1 Department of Computer Science, Courant Institute of Mathematical Sciences, New York University, 251 Mercer St, New York, 10012 USA; 2 Department of Biology, Center for Genomics and Systems Biology, New York University, 12 Waverly Pl, New York, 10003 USA

**Keywords:** Gene regulatory networks, Machine learning, Transcriptional regulation, Non homogeneous ensemble

## Abstract

This study evaluates both a variety of existing base causal inference methods and a variety of ensemble methods. We show that: (i) base network inference methods vary in their performance across different datasets, so a method that works poorly on one dataset may work well on another; (ii) a non-homogeneous ensemble method in the form of a Naive Bayes classifier leads overall to as good or better results than using the best single base method or any other ensemble method; (iii) for the best results, the ensemble method should integrate all methods that satisfy a statistical test of normality on training data. The resulting ensemble model *EnsInfer* easily integrates all kinds of RNA-seq data as well as new and existing inference methods. The paper categorizes and reviews state-of-the-art underlying methods, describes the *EnsInfer* ensemble approach in detail, and presents experimental results. The source code and data used will be made available to the community upon publication.

**Supplementary Information:**

The online version contains supplementary material available at 10.1186/s12859-023-05231-1.

## Introduction

### Network inference

A gene regulatory network (GRN) consists of molecular regulators (including DNA segments, messenger RNAs, and transcription factors) in a cell and the causal links between regulators and gene targets. Causality here means that the regulator influences the RNA expression of the gene target. Network inference is the problem of identifying such causal links. In machine learning terms, since the set of regulator genes and target genes are given, the network inference problem can be viewed as a binary classification task to determine whether or not a potential regulatory edge between any pair of regulator and target gene exists.

Because network inference facilitates the understanding of the biological systems at the level of molecular interactions, it potentially enables the designed repression or enhancement of groups of molecules. This has applications ranging from drug design and medical treatment to the reduced use of fertilizer in agriculture. Accurate network inference and functional validation is an ongoing challenge for systems biology. Over the past two decade, numerous gene regulatory network inference technologies have been proposed to tackle this problem from different perspectives [1–6].

### Individual methods feed an ensemble method

Pratapa et al. [7] presented a framework called BEELINE to evaluate state-of-the-art network inference algorithms. The vast majority of the inference algorithms including the ones we are going to incorporate into the ensemble approach can be roughly categorized into three types:

1. Pairwise correlation models make use of various kinds of correlations between a target gene’s expression and potentially causal transcription factor expressions. PPCOR [8] computes the partial and semi-partial correlation coefficients for each gene pair. LEAP [9] calculates the Pearson coefficient of each gene pair in a time-series background that considers a time-delay in regulatory response. PIDC [10] looks at the distribution of the gene expression and calculates the gene pair-wise mutual information between distributions. SCRIBE [11] also looks at mutual information between gene expression distributions, and, like LEAP, considers time-lagged correlation in time-series data. Finally, there is correlation on any set of steady-state data.
2. Tree-based models use random forests (or their close variants) to predict the gene expression of each target gene based on the expression of regulator genes (transcription factors). Such models then use feature importance to determine the weight of each regulator-target interaction. High weights correspond to regulatory edges. Examples include GENIE3 [2], a faster alternative GRNBoost2 [12], and the inference method OutPredict [13]. OutPredict also takes prior information (e.g., binding data) into account during training and test.
3. Ordinary differential equation (ODE)-based regression approaches model the target gene expression as a dependent on the time derivative of the expression of regulatory genes. Inferelator [1] is a regularized regression model that focuses on feature selection. Its latest iteration, Inferelator 3.0 [14], makes use of single cell data to learn regulatory networks. SCODE [3] is a direct application of fast ODE-based regression. SINCERITIES [15] utilizes Kolmogorov-Smirnov test-based ridge regression. GRISLI [16] is an ODE solver that accounts for gene expression velocity.

The BEELINE benchmark of 12 different inference algorithms showed that while some algorithms generally perform better than others, there is no definitive best solution that can be applied to all datasets. Our approach complements theirs: in addition to studying the performance of individual algorithms (including some promising ones that they did not study), we show that an ensemble method that we call *EnsInfer* can obtain as good or better results than any single method and improves upon previous ensemble methods [17–19]. In vision and language applications, some work, such as [20, 21], uses clustering based ensemble on large data to create balanced sets which are then sent to distinct learners. In addition to showing the benefits of combining multiple inference methods, our pipeline also provides a practical combination strategy.

## Materials and methods

### Underlying network inference algorithms

Here we introduce the inference algorithms we used in this ensemble workflow. Our workflow and the open-source code we provide allows the easy incorporation of new inference algorithms.

### Experimental setup: the data

All level 1 network inference algorithms take gene expression level data as input, there are two main sources for these data: synthetic data generated by simulation software with a given regulatory network or transcriptome-wide RNA sequencing (RNA-seq) data from living organisms. These data can be measured in a temporal manner to constitute time-series data or measured in temporally unrelated discrete states to constitute steady-state data. RNA-seq data can also be classified into two categories: bulk RNA-seq data which is obtained using all cells inside a sample tissue and single cell RNA-seq data which examines the transcriptome information of a single cell [22]. Details about the gene expression datasets used in our experiments are listed below:

1. Synthetic data from the DREAM3 and DREAM4 in silico challenges consists of ten datasets each with 100 genes and varying regulatory network structures [23, 24]. The gene expression data was generated by GeneNetWeaver, the software which provided data for the DREAM3 and DREAM4 challenges. Simulation settings were kept as the default DREAM4 challenge settings except that we generated five different time intervals between data points: 10 min, 20 min, 25 min, 50 min (default value), and 100 min. The benefit of using this synthetic data is that the underlying network is precisely known by construction.
2. Bacterial experimental RNA-seq data from *B. subtilis* (bulk RNA) containing 4218 genes and 239 TFs. The training and testing sets came from a network consisting of 154 TFs and 3144 regulatory edges [25].
3. Plant experimental RNA-seq data (bulk RNA, time-series) from Arabidopsis shoot tissue consisting of 2286 genes and 263 transcription factors (TFs). Both the training and testing sets came from a network consisting of 29 TFs and 4247 regulatory edges [26].
4. Mouse Embryonic Stem Cell (mESC) experimental single-cell RNA-seq data. containing 500 genes and 47 TFs. The training and testing sets came from a functional interaction network consisting of 47 TFs and 3226 regulatory edges [27].
5. Human Embryonic Stem Cell (hESC) experimental single-cell RNA-seq data containing 1115 genes and 130 TFs. The training and testing sets came from a ChiP-Seq network consisting of 130 TFs and 3144 regulatory edges [28].

In this work, we have focused on either temporal time-series bulk RNA-seq or single cell RNA-seq data for which pseudo-time information is available. One reason is that some of the inference algorithms in the BEELINE framework require temporal information input. The other is the well known epistemiological reason: steady state data gives simultaneous correlation information, but does not clarify the causal relationship. By contrast, because causation moves forward in time, time series datasets are more useful for causal network inference.

### Ensemble approach

Because one single inference method may not (and, in fact, does not) suit all scenarios, we propose *EnsInfer*, an ensemble approach to the network inference problem: each individual network inference method will work as a first level learning algorithm that gives a set of predictions from the gene expression input. Then we train a second-level ensemble learning algorithm that combines results from those first level learners. As first level inference methods are all different from each other, this forms a heterogeneous stacking ensemble process [29, 30]. The end goal is the binary classification task of determining whether or not a potential regulating edge from transcription factor gene to target gene exists.

**Fig. 1:**
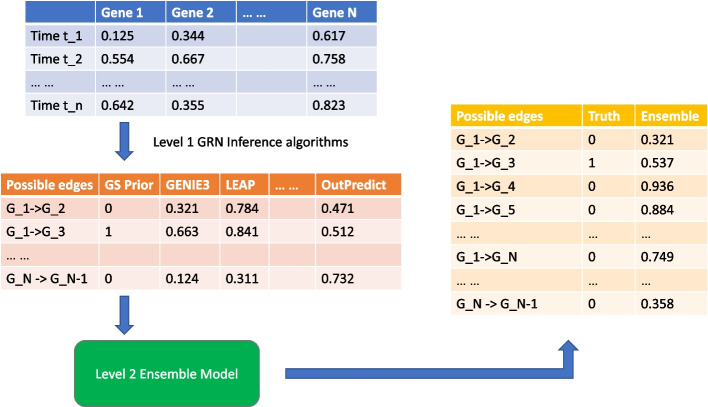
A diagram showing how *EnsInfer works * (i) All level 1 network inference algorithms are executed using time-series expression data. (ii) Every level 1 inference method assigns confidence values to all possible edges in the network. All the outputs are then curated into a tabular form with each algorithm’s prediction as a feature column. (iii) The outputs of the level 1 inference methods are then used as input data for the level 2 ensemble model, which makes predictions of regulatory edges

Thus, base network inference methods such as GENIE3 or Inferelator will work as Level 1 inference methods and individually predict whether some transcription factor *TF* regulates some target gene *g* by giving each possible edge a confidence score. The resulting edge predictions of all the level 1 inference methods can be fed into the second level ensemble learner. Previous ensemble approaches include a voting method [17, 18], but other approaches have been used for other applications: a random forest classifier, or a Naive Bayesian classifier. The pipeline is shown in Fig. 1.

Each level 1 inference method infers regulation based on all the given gene expression data. By contrast, the ensemble learner takes a training set consisting of a randomly chosen subset of regulators from gold standard (normally, experimentally verified present/absent) edges and creates a model whose input is the confidence score output of each level 1 inference method and whose output is a prediction about whether each potential edge regulates or not. One thing to note is that, for the sake of consistency across different methods, we use the confidence scores on all regulatory edges of each level 1 inference method not just the highly confident edges. This benefits the level 2 ensemble efforts, because all information inferred from level 1 methods is preserved for level 2 models.

The ensemble method uses this model and the outputs of the level 1 inference methods to predict for each transcription factor in the test set, whether a given possible edge leaving that transcription factor corresponds to a true regulatory relation. This process translates well to real world applications, where *EnsInfer* learns from the known regulatory relations within an organism or tissue structure, and makes predictions for untested transcription factors.

We evaluated eight different models to function as level 2 ensemble models using synthetic data. Those models include: voting [17], logistic regression, logistic regression with stochastic gradient descent (SGD), Naive Bayes with a Gaussian kernel, support vector machines, k-nearest neighbors, random forest, adaptive boost trees, and XGBoost [31]. All models except XGBoost are provided by the scikit-learn python package [32]. We used a separate DREAM4 dataset with 100 genes to perform hyper-parameter tuning for all level 2 ensemble models. For each of the tunable ensemble models, a discrete set of hyper-parameter combinations spanned by the common selections of core model parameters were cross-validated on this DREAM4 dataset For each method, the best performing hyper-parameter combination was used for the later level 2 comparison experiments. Details about the hyper-parameter grid search and resulting best parameter settings for each model can be found in Additional file 1: Table S1.

We compare the area under precision-recall curve on the test data of the ensemble learner against that of the level 1 inference methods that have access to the same training data.

###  Algorithmic workflow of the ensemble approach

All underlying inference algorithms were executed through the BEELINE framework proposed by [7] to which we added OutPredict and Inferelator which weren’t included in the original BEELINE package.

**Fig. 2:**
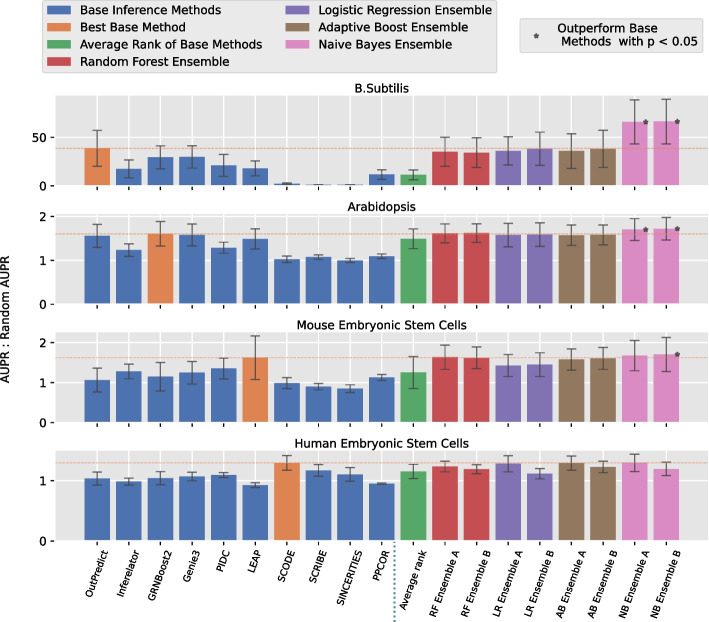
The performance of various network inference methods on three different species, from top to bottom: a *B. subtilis* gene regulatory network using bulk RNA-seq expression data [25]; an Arabidopsis network using bulk RNA-seq expression data [26]; a mouse Embryonic Stem Cell functional interaction network using single cell RNA-seq data [27] and a human Embryonic Stem Cell ChIP-seq network using single cell RNA-seq expression data [28]. Inference performance was measured using the ratio of the AUPRC of each inference method divided by that of a random predictor. Gold standard priors from each of the three species were split into a random 2:1 training/testing configuration. Ensemble models along with base inference methods that are able to incorporate prior information were trained using training gold standard priors. Then all inference results were applied using the testing subset of the gold standard data yielding an AUPRC ratio using 20 random training/testing split setups. The mean AUPRC ratio of each method on the test data among these 20 experiments is represented in the bar chart. All four ensemble models were evaluated here with base inference methods, and each of them was trained with the three worst performing base inference methods in training set (see the A series histogram) or without the three worst performing base inference methods (B series histogram). Asterisks indicate a statistically significant (*p*-value below 0.05 in non-parametric paired tests) improvement compared to the best level 1 inference method (and compared to the average ranking approach). Overall, Naive Bayes performs best, but in some cases Adaptive Boosting and Random Forests do almost as well

The confidence scores of the underlying algorithms for each potential edge in the regulatory network became inputs to the level 2 ensemble model, as illustrated in Fig. 1. To compare the performance of different inference methods, we use Area Under the Precision-Recall Curve (AUPRC) as the primary metric in all experiments. The reason for choosing AUPRC is that experimentalists can choose a high confidence cutoff to identify the most likely causal transcription factors for a given target gene. A comprehensive summary of the results can be found in Tables 1 and 2 for experiments on the DREAM in silico datasets and Fig. 2 for the three real world species.

**Table 1:** Summary of the different gene regulatory networks used in 10 DREAM simulation experiments

Number of edges	10 minutes intervals		20 minutes intervals		25 minutes intervals		50 minutes intervals		100 minutes intervals	
	Best model in training	Best model in testing	Best model in training	Best model in testing	Best model in training	Best model in testing	Best model in training	Best model in testing	Best model in training	Best model in testing
125	SCRIBE	LEAP	OutPredict	Inferelator	OutPredict	GRISLI	OutPredict	OutPredict	OutPredict	OutPredict
119	OutPredict	OutPredict	Inferelator	Inferelator	Inferelator	OutPredict	OutPredict	OutPredict	OutPredict	Inferelator
166	Inferelator	Inferelator	Inferelator	Inferelator	Inferelator	Inferelator	Inferelator	Inferelator	OutPredict	Inferelator
389	Inferelator	Inferelator	Inferelator	Inferelator	Inferelator	Inferelator	Inferelator	Inferelator	Inferelator	OutPredict
551	Inferelator	Inferelator	Inferelator	Inferelator	Inferelator	Inferelator	Inferelator	Inferelator	Inferelator	Inferelator
176	Inferelator	LEAP	Inferelator	GRNVBEM	Inferelator	Inferelator	Inferelator	Inferelator	OutPredict	PIDC
249	Inferelator	GRNBoost	Inferelator	GRNBoost	Inferelator	Genie3	OutPredict	OutPredict	Inferelator	Inferelator
195	Inferelator	Inferelator	Inferelator	Inferelator	Inferelator	Inferelator	Inferelator	Inferelator	Inferelator	Inferelator
211	Inferelator	Inferelator	Inferelator	SCRIBE	Inferelator	Inferelator	Inferelator	Inferelator	OutPredict	Genie3
193	Genie3	Inferelator	Inferelator	Inferelator	Inferelator	Inferelator	Inferelator	Inferelator	OutPredict	Inferelator

The best level 1 inference methods for five different time interval settings (measurements every 10 min, every 20 min, every 25 min, 50 min, 100 min) for the time-series. Often, the best model on the training set is also the best model on the test set. Qualitatively, when the intervals are shorter, differential equation style methods are best. When the intervals become larger, random forest methods are often superior

**Table 2:** Relative performance of different ensemble methods using all level 1 inference methods’ results (i.e., regardless of kurtosis) as ensemble inputs and the same models while only using level 1 inference methods’ results with positive kurtosis, marked by plus signs (corresponding to positive kurtosis)

Ensemble model	Interval between time-series data points				
	10 min	20 min	25 min	50 min	100 min
Logistic regression	1.11 ± 0.45	0.99 ± 0.21	0.92 ± 0.34	1.33 ± 0.56	**1.25 ± 0.44**
Logistic regression$^+$	1.13 ± 0.48	1.12 ± 0.43	0.95 ± 0.35	1.31 ± 0.54	1.24 ± 0.46
Logistic regression with SGD	0.25 ± 0.06	0.4 ± 0.47	0.2 ± 0.11	0.2 ± 0.14	0.26 ± 0.19
Logistic regression with SGD$^+$	0.71 ± 0.23	0.63 ± 0.19	0.54 ± 0.08	0.55 ± 0.15	0.57 ± 0.21
Naive Bayes	0.83 ± 0.23	0.71 ± 0.17	0.65 ± 0.15	0.67 ± 0.16	0.68 ± 0.26
Naive Bayes$^+$	**1.33 ± 0.58**	1.09 ± 0.26	**1.19 ± 0.5**	1.14 ± 0.48	1.13 ± 0.35
Support vector machine	0.21 ± 0.09	0.29 ± 0.14	0.26 ± 0.09	0.25 ± 0.15	0.26 ± 0.22
Support vector machine$^+$	0.47 ± 0.2	0.51 ± 0.19	0.43 ± 0.12	0.86 ± 0.6	0.47 ± 0.25
K-nearest neighbors	0.59 ± 0.24	0.45 ± 0.22	0.51 ± 0.2	0.39 ± 0.34	0.57 ± 0.41
K-nearest neighbors$^+$	0.58 ± 0.23	0.68 ± 0.54	0.71 ± 0.47	0.6 ± 0.46	0.65 ± 0.31
Random forest	1.12 ± 0.56	0.9 ± 0.21	0.99 ± 0.29	**1.73 ± 1.03**	1.09 ± 0.38
Random forest$^+$	1.1 ± 0.51	0.97 ± 0.42	1.04 ± 0.37	1.73 ± 1.1	1.08 ± 0.34
Adaptive boosting	1.12 ± 0.5	**1.13 ± 0.76**	0.73 ± 0.26	1.27 ± 0.65	0.95 ± 0.46
Adaptive boosting$^+$	1.07 ± 0.43	1.11 ± 0.65	0.82 ± 0.27	1.3 ± 0.7	1.03 ± 0.49
XGBoost	0.65 ± 0.34	0.56 ± 0.16	0.61 ± 0.28	1.35 ± 0.86	0.66 ± 0.24
XGBoost$^+$	0.6 ± 0.25	0.58 ± 0.23	0.78 ± 0.63	1.31 ± 0.86	0.67 ± 0.26
Best level 1 method in training	0.88 ± 0.23	0.88 ± 0.2	0.88 ± 0.24	1 ± 0	0.81 ± 0.22
Best level 1 method evaluated on all samples	0.93 ± 0.26	1.11 ± 0.58	1.08 ± 0.34	1.03 ± 0.49	0.95 ± 0.44
Average rank of level 1 methods	0.95 ± 0.42	0.88 ± 0.52	0.78 ± 0.39	0.69 ± 0.16	0.61 ± 0.22

Experiments were done across five different DREAM simulation settings for time-series intervals. The performance metric is the ratio of AUPRC score of the ensemble method compared to that of the best performing level 1 inference method in testing. Each cell is the mean value and standard deviation across the ten DREAM datasets with varying regulatory networks. The bold number in each column is the best performing value in that time interval setting. Logistic regression, random forest and adaptive boosting models yielded top level inference performance among all ensemble options when there was no filtering based on kurtosis. With kurtosis filtering, the Naive Bayes and logistic regression approaches yield the best overall results while performances from random forest and adaptive boosting are still competitive

For the in silico DREAM datasets, the underlying gold standard priors that define each regulatory network were divided into a 2:1 training/testing split, so there were twice as many regulators in training as in testing. Because the split was done with respect to the regulators, the training and testing sets share no common transcription factors. We believe splitting based on transcription factors is the correct approach, because experimental assays commonly over-express or repress particular transcription factors. The practical goal is that if a species has some TFs with experimentally validated edges, then edges from untested TFs can be inferred.

For each dataset, we first applied 11 base level inference methods to the training data both to determine a promising single method to apply to the test data and as an input to the construction of the ensemble model. Out of the 12 methods included in BEELINE, SINCERITIES, SINGE and SCNS either produced no output or exceeded the time limit of one week for one or more of the datasets. We applied those individual level 1 inference methods (not only the most promising ones from the training data) as well as the level 2 non homogeneous ensemble models to the test set.

To assess ensemble models, we compared them with one another and with the best level 1 inference methods in both training and testing evaluations. As [17] have pointed out for the DREAM challenge, one simple yet (in DREAM at least) effective way to integrate multiple inference results is to rank potential edges according to their average rank given by all inference methods. We will also include this “community” method as a reference point for our ensemble models.

The experiments on the DREAM in silico datasets focused on three objectives: (i) for each dataset, how well did the level 1 inference methods that performed best on the training set perform on the test set? (ii) how well did the ensemble learners perform on the test set? (iii) how did the level 1 inference method that performed best on the test set compare to the level 2 ensemble models? Note that the comparison of (iii) is unfair to the ensemble models, because there is no way to know a priori which level 1 inference method will perform best on a given test set, so choosing the best one gives an unfair advantage to the level 1 inference methods.

On the experimental datasets from real world species, similarly, four level 1 methods from BEELINE: GRNVBEM, GRISLI, SINGE and SCNS were not able to produce proper inference results due to time or memory constraints on the larger datasets (e.g. they did not finish after a week), hence were not included in the ensemble approach. We then applied the best performing level 2 ensemble models from the DREAM experiments to the available 10 base level inference methods. Furthermore, we varied the input to the level 2 ensemble models by including or excluding the results from the three most poorly performing level 1 inference methods.

## Results

### Base inference method performance

On the DREAM datasets, the performance of the algorithms featured in the BEELINE framework is consistent with the original paper [7]. GENIE3, GRNBOOST and PIDC performed the best among the algorithms the BEELINE authors tested. As it happened, the methods we added to the framework (Inferelator and OutPredict) outperformed those methods in many cases. Nevertheless, no individual level 1 inference method dominated the others, as seen in Table 1. We also note that while the best level 1 inference methods in training is often the best algorithm in testing, that is not always the case.

### Ensemble performance

The Naive Bayes model we used works on the assumption that the likelihood distribution of edge presence is Gaussian-like with respect to any given input’s confidence score. On the DREAM datasets, two of the eleven level 1 inference methods (GRISLI and PIDC) produced outputs that did not have a Gaussian like distribution (reflected as a negative kurtosis). We therefore experimented using all level 1 inference methods as input as well as using only the level 1 inference methods whose output distribution has positive kurtosis as inputs to better accommodate Naive Bayes model. The combined results are presented in Table 2. For most ensemble methods, using all 11 level 1 inference methods versus using 9 does not change the result. However, for Naive Bayes and Logistic Regression with Stochastic Gradient Descent, eliminating those level 1 inference methods that produce non-Gaussian like output helps. In fact, Naive Bayes is overall the winner across all tested models and configurations when the input is limited by the positive kurtosis filter. While logistic regression, random forest and adaptive boosting also performed favorably compared to the best performing level 1 inference methods in training as well as compared to the average rank of level 1 inference methods of [17].

Hence, for real world experimental datasets, the four models: logistic regression, Naive Bayes, random forest and adaptive boosting were selected as level 2 ensemble models for analysis (see Fig. 2). Here the likelihood distributions of all results from the level 1 inference methods have a positive kurtosis measure, so all 10 of them were utilized for the level 2 ensemble methods.

Figure 2 shows that

- The Naive Bayes approach on inputs having positive kurtosis outperforms the other three ensemble method, so our system *EnsInfer* uses Naive Bayes as the default option.
- Including results from weak learners has a marginal impact (sometimes positive and sometimes negative) on the final ensemble performance. For the sake of simplicity, therefore, *EnsInfer* includes inputs from all available inference methods having positive kurtosis, even the weak ones.

**Fig. 3:**
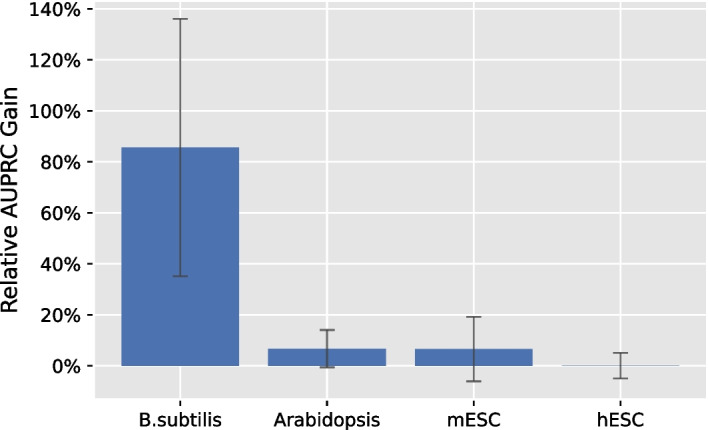
The AUPRC improvement of the Naive Bayes ensemble model (restricting inputs to those with positive kurtosis, but including weak learners) compared to the single best base inference method. In the *B. subtilis* and Arabidopsis datasets the improvement had *p*-value $< 0.05$ using a non-parametric paired test [33]. The test should be paired, because the same set of training and testing splits were used for every method. In the human dataset, the ensemble method was about equal to the best base inference method. As noted in the text, the best base method cannot be known a priori, so these comparisons understate the advantage of the ensemble method

Figure 3 shows that the Naive Bayes ensemble approach significantly (*p*-value < 0.05) outperformed the best level 1 method on *B. subtilis* and Arabidopsis. The ensemble method with all level 1 methods still had an advantage in mESC data although the performance gain was less statistically significant with *p*-value of 0.133. To calculate the *p*-value, we conservatively chose a non-parametric paired resampling approach [33] because we did not want to assume any particular distribution on the data. We used a paired test because we measured the AUPRC gain for each training/test split. (That is, the set of training/testing splits were established randomly and initially. Then, for the numerical experiments, each method used that set.) In the hESC case, the Naive Bayes ensemble method achieved approximately the same level of performance as the best level 1 method on the test set. As noted above, the best level 1 inference method for the test set cannot be known a priori (and not even looking at each method’s performance in training), so using an ensemble method gives high performance without having to know which level 1 inference method is best. The Naive Bayes approach also consistently outperformed the average voting ensemble approach [17].

*EnsInfer*: Compared to running a single inference method, the ensemble approach requires an amount of computation resources equal to the sum of the time to run all base inference algorithms, plus the ensemble effort itself. However, all base inference methods can be executed in parallel, so the wall clock time of executing level 1 inference process is just the time of the slowest method which often is also single threaded. The level 2 ensemble effort itself is less than 1/10 the time of the slowest base method as shown in Additional file 2: Table S2. We can therefore conclude that *EnsInfer*’s wall clock time is close to that of the slowest base inference method.

## Discussion

Consistently with [7], we find that no one inference method is best for all datasets tested in our study. However, a Naive Bayes level 2 ensemble model built from level 1 inference methods having positive kurtosis holds great promise as a general ensemble network inference approach and is thus the basis of *EnsInfer*. Naive Bayes may work better than more sophisticated Bayesian methods, because at the core of the Bayesian method, we need to estimate the likelihood distribution of *p*(*x*|*e*) where *x* is the score given by a level 1 inference method and *e* is the existence of an edge. Since this generative process varies dramatically across different datasets and inference methods, the Gaussian assumption used by Naive Bayes is as good as any and keeps the model simple.

Please note, however, that there are cases when Naive Bayes does not improve on the best single inference method. This happens primarily when the results are little better than random. For example, the inferred regulatory networks from single-cell human embryonic stem cell data from [7] was barely better than random using any base method in BEELINE. The ensemble does not improve that.

Naive Bayes works particularly well in a sparse data environment, which is often the case when experimental data is hard to come by. For example, there are only 29 experimentally validated transcription factors for Arabidopsis and 154 for *B. subtilis*. Another point in favor of Naive Bayes is that the the size of the feature space (the number of outputs of the level 1 inference methods) is small. If the training dataset and feature space were larger, Random Forest-based approaches might do better. Our current investigation used roughly a dozen level 1 inference methods. Other promising new inference ones could be added such as BiXGBoost and DeepSEM [4, 5]. A level 2 ensemble method might potentially require a feature selection step if many more inference algorithms were included.

## Conclusion

The main overall benefit of the ensemble method *EnsInfer* is its robust and flexible nature. Instead of picking a network inference method and hoping that it will perform well on a dataset, *EnsInfer* uses a combination of state-of-the-art inference approaches and combines them using a simple Naive Bayes ensemble model. Because the ensemble approach essentially turns all the predictions from different inference algorithms into priors about each edge in the network, *EnsInfer* easily allows the integration of diverse kinds of data (e.g. bulk RNA-seq, single cell RNA-seq) as well as new inference methods.

## Supplementary information

**Additional file 1: Table S1.** Parameter search space for all ensemble methods used in our experiments, and the optimal parameters obtained from tuning on a DREAM dataset.

**Additional file 2: Table S2.** Execution time of various inference algorithms and ensemble methods used in this research. Time was measured for the mESC, hESC, arabidopsis and *B. subtilis* dataset, on an Ubuntu 20.04 system with AMD Ryzen™ 9 5900X CPU.

## Data Availability

All experimental data and source code for the ensemble process can be found at our Github repository: https://github.com/IcyFermion/network_inference_ensemble
